# Patterns of Self-Reported Unmet Healthcare Needs Among Canadians Aged 15 Years and Older in 2001: A Descriptive Cross-Sectional Analysis

**DOI:** 10.7759/cureus.97678

**Published:** 2025-11-24

**Authors:** Echezona O Ukaejiofo, Ifunanya C Modebelu, Oyewale Fakoya, Mofiyinfoluwa Fagbemi, Oreoluwa Sonaike, Victor Oyelude

**Affiliations:** 1 General Practice, College of Medicine, University of Nigeria, Enugu, NGA; 2 General Practice, College of Medicine, University of Nigeria Teaching Hospital, Enugu, NGA; 3 Medicine, Obafemi Awolowo College of Health Science, Olabisi Onabanjo University, Sagamu, NGA; 4 Family Medicine, All Saints University School of Medicine, Roseau, DMA; 5 Family Medicine, College of Medicine, Lagos State University, Lagos, NGA; 6 Family Medicine, MedCare Clinics, Niagara Falls, CAN

**Keywords:** canada, chronic disease, healthcare access, service availability, unmet healthcare needs, waiting times

## Abstract

Background: Access to timely and adequate healthcare remains a major public health concern, even in countries with universal health coverage such as Canada. Unmet healthcare needs, particularly among individuals with chronic conditions, highlight significant gaps in service delivery and equity.

Objective: This study aimed to describe the distribution and relative proportions of self-reported reasons for unmet healthcare needs among Canadians aged 15 years and older.

Methods: A descriptive cross-sectional analysis was conducted using secondary data from Statistics Canada's Open Government Portal (Table 13-10-0692, 2015). The dataset included aggregated national estimates representing a total study population of 2,546,977 individuals aged 15 years and above. Only valid percentage-based data were analyzed, excluding flagged or unreliable records. Descriptive statistics and visual summaries were used to identify the most common barriers.

Results: Among the study population (n=2,546,977), 36 valid aggregated observations, long waiting times (n=1,044,318; 49.4%), and service unavailability (n=773,451; 36.6%) were the leading reasons for unmet healthcare needs. Other reported barriers included perceived inadequacy of care (n=157,714; 7.5%) and personal constraints such as being too busy (n=77,600; 3.7%).

Conclusion: Prolonged waiting times and limited service availability remain the most critical barriers to healthcare access in Canada, particularly affecting individuals with chronic illnesses. These findings should be interpreted with caution, as several data points in the Statistics Canada file were flagged for limited reliability and subsequently excluded, which may narrow the completeness of the reported barriers.

## Introduction

Ensuring that healthcare is both accessible and affordable in a timely manner is fundamental to improving population health outcomes [1]. While unmet healthcare needs are recognized internationally, the present study focuses specifically on the Canadian context and does not include direct international data comparisons. Even in industrialized countries with universal or even close to universal services, huge masses of individuals testify to having unmet healthcare requirements, i.e., they needed healthcare, but were not able to access it [2]. In Canada, some population surveys have documented the fact that a nontrivial percentage of the adults in the country are facing unmet needs that reflect a gap in the health system of providing care on an equal basis [3]. These needs remain unmet due to complex and multifaceted issues such as cost barriers, limited service availability, long waiting times, transportation challenges, and more that are believed to be caused by economic, structural, individual, and system navigation barriers [4]. The given patterns are especially important to comprehend with reference to chronic diseases, such as diabetes, hypertension, or mental issues, when constant access to care is the most important factor in the prevention of complications [5].

Based on established evidence in the literature, cost barriers are one of the vivid dimensions as basic physician and hospital services can be publicly covered because there are still out-of-pocket expenses exposing the patient to medications, visits to a specialist, or an assistive device [6]. Cost-related nonadherence to prescription drugs is a phenomenon common in Canada, with one out of 10 Canadians missing doses or neglecting drugs due to lack of affordability, particularly among the poorer population with chronic conditions or the less affluent [7,8]. 

The other dimension is service unavailability based on geographical or time factors [9]. In rural or remote locations, the clinic or specialists might not be available at the time that a patient requires it, some of the services might be unavailable in the area, and the patient has to either travel or wait [10]. In line with that, long waiting times also continue to be a problem in Canada: when it comes to specialist consultations or diagnostic imaging, waiting times are frequently cited as the cause of unmet need among Canadians [11,12].

The problems of providers are also topical: some of them give reasons related to fears, dissatisfaction with the attitude of clinicians, or the belief that care is inadequate [13]. The language and communication barriers are particularly applicable in the case of new individuals and non-English/French speakers: immigrants may find it hard to navigate through the health system and medical care, comprehend care guidelines, and communicate their needs [14]. 

There are also personal or family-related limitations on care-seeking: caregivers, workers, or childcare obligations can delay or entirely avoid appointments [15]. Excessive barriers are also increased by transportation challenges, particularly in rural areas or low-income communities, where limited access to reliable transport and longer travel times can delay or prevent individuals from reaching clinics or specialists [16]. Finally, issues with navigating health systems, such as a lack of knowledge about the service needed, the place to visit, and the time to book an appointment, are also a frequently reported reason among individuals with unmet needs [17].

These barriers are particularly impactful in the framework of diabetes, hypertension, and mental health/psychiatry [18]. Treatment of chronic diseases requires frequent follow-up, adherence to medications, laboratory tests, and access to specialist treatment (e.g., endocrinologists, nephrologists, psychiatrists) [19]. Patients may not receive necessary monitoring or medication modifications when there is an intervening factor of cost, distance, waiting times, or communication barriers, since this will increase the risk of complications such as cardiovascular disease or failure of the kidneys [20]. The objective of the study is to describe the distribution and relative proportions of self-reported reasons for unmet healthcare needs among Canadians aged 15 years and older in 2001. It will also identify the most frequently reported barriers, such as long waiting times, service unavailability, and perceived inadequacy of care, using nationally representative data from Statistics Canada [21].

## Materials and methods

Study design and data source

This study adopted a descriptive cross-sectional design using secondary data obtained from the Open Government Portal (Government of Canada). The dataset was identified as Series Issue ID: Table 13-10-0692 - Reasons for Self-Reported Unmet Needs for Health Care Services, Household Population Aged 15 and Over, Canada [21]. The dataset provides aggregated national estimates from the 2001 health survey, capturing information from Canadians aged 15 years and older on reasons for unmet healthcare needs. Data collection and dissemination adhered to Statistics Canada's national standards and survey protocols. The dataset offers a nationally representative overview of the main barriers to healthcare access among the Canadian household population.

The dataset was provided in an aggregated format, and the original survey instrument, microdata, and weighting procedures were not accessible through Table 13-10-0692. Consequently, details such as survey question wording, sampling weights, and variable construction could not be reported. "E"-flagged estimates were retained but interpreted with caution in accordance with Statistics Canada guidance. Aggregation followed the structure of the dataset, which grouped responses into predefined categories without further demographic or clinical breakdowns. These limitations reduce the reproducibility of the analysis but are inherent to the publicly available data format.

Study population

The study population consisted of Canadian residents aged 15 years and older who participated in the 2001 national health survey and reported at least one instance of an unmet healthcare need. The population base excluded individuals living in institutions and on Indigenous reserves, in accordance with Statistics Canada's survey design. The final aggregated data represent the household population and provide a national-level summary of Canadians who experienced difficulties in accessing healthcare services.

Variables and measures

The principal variable analyzed was the reason for self-reported unmet healthcare needs. The dataset initially contained 14 categories representing various barriers, including long waiting times, service unavailability, cost-related issues, transportation difficulties, perceived inadequacy of care, and other personal or systemic constraints. Each reason was accompanied by multiple statistical measures, such as the number of persons, confidence intervals, coefficients of variation, and percentage estimates. For simplicity and clarity, this study retained only two core measures, that is, number of persons and percentage, since these provide the most direct and interpretable indicators of the distribution of reported barriers across the population.

Statistical analysis

Descriptive statistical methods were used to summarize and interpret the data. The total number of respondents represented in the analysis was 2,546,977, corresponding to all individuals who reported at least one unmet healthcare need. The frequency and percentage distribution of each unmet need category were examined to identify the most commonly cited reasons. The percentage values were derived from aggregated weighted estimates provided in the Statistics Canada dataset rather than being calculated directly from the total study population. Specifically, duplicate entries under each reason category were combined, and corresponding percentage measures were collapsed and summed using Stata to generate aggregated weighted proportions consistent with the official dataset structure. A descriptive summary table was generated to present the number of persons and corresponding percentages for each barrier. All analyses were performed using Stata Version 18 (StataCorp LLC, College Station, Texas, United States).

Missing data

The dataset did not contain conventional missing numerical values; however, certain categories were flagged by Statistics Canada for data reliability concerns. Entries labeled "E" indicated estimates to be used with caution, while those labeled "F" were considered too unreliable to be published. In this study, all categories marked "F" were excluded since they did not contain valid numerical or percentage data. The removed categories included "Cost", "Personal or family responsibilities", "Language problems", and "Decided not to seek care". After excluding these unreliable entries and retaining valid measures, the retained observations corresponded to an estimated total of 2,546,977 Canadians, providing a credible basis for descriptive interpretation.

Ethical considerations

This study was based on publicly available, anonymized secondary data obtained from the Open Government Canada platform. The dataset contains aggregated national estimates with no personally identifiable information. Therefore, formal ethical approval was not required under institutional or national research ethics guidelines. The use of the dataset fully complies with Statistics Canada's Open Data License and adheres to the principles outlined in the Tri-Council Policy Statement: Ethical Conduct for Research Involving Humans (TCPS 2).

## Results

Table 1 presents the distribution of self-reported reasons for unmet healthcare needs among Canadians aged 15 years and older based on aggregated 2001 national survey data. The analysis was conducted on an estimated population of 2,546,977 individuals who reported experiencing at least one barrier to accessing health services. The number of persons (n) and the corresponding percentages represent aggregated weighted estimates derived from Statistics Canada's published data. Therefore, the percentages do not result from dividing the subgroup totals by the overall study population but rather reflect survey-weighted proportions that account for population design and category aggregation. The findings highlight the primary factors contributing to unmet healthcare needs within the Canadian population and offer insight into the relative prominence of system-level and personal barriers.

**Table 1:** Distribution of self-reported reasons for unmet healthcare needs among the Canadian household population aged 15 years and older (Canada, 2001) Percentages represent aggregated weighted estimates obtained from Statistics Canada's Table 13-10-0692. These values are not simple ratios of the raw counts but are based on population-weighted proportions reflecting the survey's complex sampling structure. The symbol "—" indicates categories excluded due to unreliable data (flagged as "F" by Statistics Canada).

Unmet healthcare need reasons	Number of persons (n)	Percent (%)
Waiting time too long	1,044,318	49.4
Service not available when needed	773,451	36.6
Other barriers	335,898	15.9
Felt service would be inadequate	157,714	7.5
Too busy	77,600	3.7
Didn't get around to it or didn't bother	65,223	3.1
Dislikes physicians or is afraid	45,929	2.2
Didn't know where to go	30,514	1.4
Transportation problems	16,330	—

The results show that long waiting time was the most commonly reported barrier to accessing healthcare, accounting for approximately 1,044,318 persons (49.4%). This finding underscores the prominence of delays within the Canadian health system as a major deterrent to timely care. The second most frequently cited reason was that services were not available when needed, reported by 773,451 persons (36.6%), suggesting that accessibility and availability of services remain substantial challenges. Other notable reasons included other barriers, cited by 335,898 persons (15.9%), reflecting a mix of less-defined but relevant structural or personal constraints. Additionally, 157,714 persons (7.5%) indicated that they felt the service would be inadequate, while 77,600 (3.7%) reported being too busy to seek care. Smaller proportions reported more personal or perceptual barriers, such as 65,223 (3.1%) who didn't get around to it or didn't bother, 45,929 (2.2%) who disliked or were afraid of physicians, and 30,514 (1.4%) who didn't know where to go for care. A minor category, transportation problems, was identified but not quantified due to being flagged as unreliable.

Overall, these findings indicate that the majority of unmet healthcare needs in Canada were attributed to system-level issues, particularly excessive waiting times and limited service availability, rather than individual-level factors such as motivation, knowledge, or fear. The distribution highlights persistent accessibility challenges within the Canadian health system, emphasizing the need for targeted interventions aimed at reducing delays and improving service availability across regions and populations.

## Discussion

The present study examined the distribution and reasons for unmet healthcare needs among Canadians aged 15 years and older, revealing persistent barriers that continue to influence access to care. The findings confirm that a substantial portion of the population experiences difficulties obtaining needed services due to factors such as long waiting times, limited service availability, and dissatisfaction with care. These outcomes echo previous studies that have documented similar access inequities in both primary and specialized healthcare settings [1-3]. Despite Canada's publicly funded system, the results emphasize that the existence of universal coverage does not necessarily translate into equitable access, particularly for individuals managing chronic diseases.

For individuals with conditions such as diabetes, hypertension, or psychiatric disorders, unmet healthcare needs have particularly serious implications. These groups require continuous and coordinated management to prevent complications, maintain stability, and reduce the likelihood of hospitalizations [5,18,19]. When care is delayed or inaccessible due to cost, waiting times, or service unavailability, patients often experience disease progression and reduced quality of life [6,8,11]. For instance, individuals with diabetes who face prolonged waiting times for follow-up appointments may miss opportunities for timely medication adjustments or monitoring of glycemic control, heightening the risk of cardiovascular and renal complications [19,20]. Similarly, hypertension management depends on regular assessment and adherence to therapy; when cost or service barriers intervene, blood pressure control deteriorates, thereby increasing the risk of stroke and heart disease [5].

In mental and psychiatric health, unmet needs are particularly damaging. Previous research has shown that barriers such as long waits for specialist care, stigma, or dissatisfaction with provider attitudes can discourage patients from seeking timely help [13,15]. This can lead to worsening symptoms, crisis episodes, and increased emergency department use [4,14]. The current findings align with this evidence, suggesting that structural and attitudinal barriers within the healthcare system continue to impede access to mental health services despite policy commitments toward equity of care. Individuals with chronic psychiatric conditions often experience intersecting disadvantages financial instability, transportation issues, or communication difficulties that compound their vulnerability to unmet needs [14,15].

Geographic disparities also contribute to unmet needs. People living in rural or remote regions are less likely to have access to nearby specialists or diagnostic services, leading to delayed care and greater reliance on emergency services [9,10]. Such patterns disproportionately affect older adults and those with chronic illnesses who require regular monitoring. Similarly, cost-related nonadherence remains a pervasive problem; despite public coverage for many physician services, out-of-pocket expenses for medications and medical devices continue to create financial barriers, especially among lower-income households [6-8]. This reinforces the idea that affordability remains a critical determinant of access, even within publicly funded systems.

Furthermore, out-of-pocket costs for medications and medical supplies directly contribute to treatment gaps in chronic illnesses such as hypertension, diabetes, and asthma by limiting patients' ability to maintain consistent therapy [6]. Even modest increases in patient cost-sharing correlate with reduced medication adherence and higher rates of adverse health events, particularly among low-income and elderly populations [6]. When individuals cannot afford essential drugs, such as antihypertensives, they often skip doses, reduce frequency, or discontinue treatment altogether, leading to poor disease control [6,7]. This cost-related nonadherence increases the likelihood of acute complications such as diabetic ketoacidosis or hypertensive crises which drive preventable emergency visits and hospitalizations, especially among those without drug insurance or with unstable income and employment [7,8]. Among Canadians with multimorbidity, 10.2% reported cost-related nonadherence, most often within respiratory and mental health condition groups [8].

Disruptions in continuity of care among patients with diabetes, hypertension, or chronic kidney disease also have substantial clinical implications. Missed HbA1c testing or blood pressure monitoring often prevents the early detection of disease progression, delaying intervention opportunities [19,20]. Persistent hyperglycemia and uncontrolled hypertension accelerate vascular injury and end-organ decline, increasing risks of myocardial infarction, stroke, heart failure, and renal failure [18]. Emerging evidence indicates that fragmented follow-up and unmet care weaken disease monitoring and contribute to the progression of chronic conditions into major drivers of cardiovascular and renal morbidity [5].

Gaps in psychiatric care, particularly for depression, anxiety, and psychotic disorders, remain a pressing public health issue with systemic and clinical repercussions. When access to mental health services is delayed, restricted, or hindered by stigma, symptoms often intensify and comorbidities increase, pushing patients to seek emergency care instead of early intervention. Consistent evidence shows that individuals with depression or anxiety often feel dismissed during clinical encounters, discouraging continued engagement and reinforcing distress [13]. Among immigrant and minority groups, stigma, cultural misunderstandings, and prolonged wait times erode trust and cause many to delay help-seeking until conditions become unmanageable [14]. Socioeconomic barriers, distance to providers, and limited service availability further compound these delays, resulting in avoidable crises and escalating healthcare costs [15]. Reducing these disparities requires anti-stigma initiatives, timely access to culturally responsive mental health services, and stronger coordination between primary, community, and acute care sectors.

Similarly, limited service access continues to affect patients with chronic conditions such as diabetes, kidney disease, and heart disease, especially in rural and remote areas where long travel distances and wait times remain common barriers [9]. Such restricted access delays diagnosis, hinders follow-up, and worsens health outcomes. For example, residents in Saskatchewan often travel several hours for specialist consultations with cardiologists or nephrologists [10], while individuals requiring dialysis or advanced diabetes care face significant physical and emotional strain due to repeated long-distance travel [19]. In Alberta's First Nations communities, insufficient access to diabetes education, specialist support, and local care facilities further limits effective disease management [18]. These geographic inequities underscore the need for improved healthcare delivery and policy interventions targeting underserved regions to ensure equitable access to chronic disease care.

Overall, the study highlights that unmet healthcare needs are not only an indicator of systemic inefficiencies but also a reflection of underlying social and economic inequalities. The consistency between these findings and prior literature suggests that addressing such barriers requires a multidimensional approach, combining structural reforms, improved service availability, and targeted strategies for chronic disease populations. Integrated care models that ensure continuity and coordination between primary and specialist services could help mitigate many of the identified access issues [5,19].

It is also important to note that the data reflect the healthcare landscape of 2001. Since then, Canada has undergone policy updates, system reforms, and technological improvements that may influence current access patterns. As a result, the findings should be interpreted as reflective of the period studied rather than the present-day system.

Strengths and limitations of the study

This study's main strength is the use of a large, nationally representative dataset of over 2.5 million individuals, offering a broad view of healthcare access across Canada. However, several limitations must be acknowledged. A key limitation was the presence of missing or flagged data, which may have affected accuracy, along with possible recall bias from self-reported responses and the inability of the cross-sectional design to establish causality. The exclusion of unreliable categories such as cost, language problems, personal responsibilities, and transportation problems may also introduce bias by underrepresenting certain barriers and limiting the completeness of the distribution of unmet healthcare needs. In addition, the dataset provides only national-level aggregated estimates and does not include demographic or clinical breakdowns (e.g., age, gender, rural vs. urban residence, or specific chronic disease status), restricting the ability to examine disparities across subgroups. Future research should use longitudinal methods to assess long-term effects, examine socioeconomic and geographic disparities, and evaluate interventions aimed at improving affordability, specialist access, and mental health integration while enhancing data quality for more reliable analysis.

## Conclusions

This study highlighted that long waiting times, service unavailability, and perceived inadequacy of care are the most common reasons for unmet healthcare needs among Canadians. These barriers are particularly concerning for individuals with chronic conditions such as diabetes, hypertension, and mental health disorders, where continuous and timely care is essential for effective management. The findings emphasize the ongoing gaps in accessibility within the Canadian health system and underscore the need for targeted strategies to reduce waiting times, improve service availability, and enhance patient confidence in care quality to ensure equitable healthcare access for all. However, because the analysis is based on data from 2001, the findings reflect the healthcare environment of that period and may not fully represent current system conditions or recent policy and technological developments.
